# Loss of “insight” into behavioral changes in ALS: Differences across cognitive profiles

**DOI:** 10.1002/brb3.2439

**Published:** 2021-12-02

**Authors:** Anna G. M. Temp, Elisabeth Kasper, Stefan Vielhaber, Judith Machts, Andreas Hermann, Stefan Teipel, Johannes Prudlo

**Affiliations:** ^1^ German Center for Neurodegenerative Diseases (DZNE) Rostock/Greifswald Rostock Germany; ^2^ Department of Neurology Rostock University Medical Center Rostock Germany; ^3^ Department of Neurology Otto‐von‐Guericke University Magdeburg Germany; ^4^ German Center for Neurodegenerative Diseases (DZNE) Magdeburg Germany; ^5^ Translational Neurodegeneration Section “Albrecht‐Kossel” Department of Neurology and Center for Transdisciplinary Neurosciences Rostock (CTNR) University Medical Center Rostock University of Rostock Rostock Germany; ^6^ Department of Psychosomatic Medicine Rostock University Medical Center Rostock Germany

**Keywords:** amyotrophic Lateral Sclerosis, apathy, behaviour, frontotemporal dementia, insight, intelligence

## Abstract

**Objective:**

Behavioral impairment occurs in amyotrophic lateral sclerosis (ALS) and ALS–fronto‐temporal dementia (ALS‐FTD). It has been proposed that ALS patients without FTD retain an awareness of their behavioral impairment while ALS‐FTD patients lose this awareness (referred to as retention vs. loss of “insight”). Loss of insight has not yet been studied across the entire ALS‐FTD spectrum; our study addresses this gap by including patients with all the ALS cognitive‐behavioral profiles.

**Methods:**

Eighty‐three ALS patients (and their informants) took part in this bicentric study involving two German recruitment sites. Patients and informants completed the *Frontal Systems Behavior Scale* covering the domains of apathy, disinhibition, and executive dysfunctioning. Patients were classified into five groups according to the Strong and Rascovsky criteria: cognitively unimpaired (ALSni), cognitively impaired without dementia (ALSci), behaviorally impaired (ALSbi), a combination of behaviorally and cognitively impaired (ALScbi), and ALS‐FTD. We applied Bayesian two‐way ANOVA to test whether there were subgroup differences regarding insight into their behavioral decline.

**Results:**

All patient subgroups experienced behavioral decline (Bayes factor > 3). Only ALS‐FTD patients lost insight into disinhibition and executive dysfunctioning. ALSbi patients exhibited worse insight than ALSni and ALSci patients (Bayes factor > 10). Evidence regarding the ALScbi patients was inconclusive. Higher IQ was associated with worse insight (Bayes factor > 3).

**Conclusions:**

Our findings provide solid support for the notion that ALS patients without dementia experience behavioral decline regardless of their cognitive‐behavioral profile and retain different levels of insight into this decline. The inverse association of premorbid verbal intelligence with insight was unexpected, leaving room for further investigation.

## Graphical abstract

Behavioral decline occurred in ALS patients, regardless of their cognitive‐behavioral profiles. Insight into behavioral decline was present in all domains for all non‐demented patient groups, but absent for ALS‐FTD patients’ disinhibition and executive dysfunction.

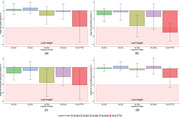

## 1 INTRODUCTION

As a multisystemic disorder, a*myotrophic lateral sclerosis* (ALS) may impair cognition and behavior alongside voluntary motor control (Benbrika et al., 2019) and forms a disease spectrum with *fronto‐temporal dementia* (ALS‐FTSD, Strong et al. 2017). Cognitive‐behavioral impairment is present in approximately 50% of ALS‐FTSD patients; this amounts to fully‐blown FTD in up to 15% of all ALS‐FTSD patients (Montuschi et al., 2015; Ringholz et al., 2005). Characteristically, cognitive impairment entails executive and/or language deficits (Beeldman et al., 2016; Benbrika et al., 2019). The most frequent symptoms of behavioral impairment on the ALS‐FTD spectrum are perseveration (40% of patients), apathy (29%), and disinhibition (26%, Raaphorst et al., 2012). Perseveration includes behavioral repetition without flexibly adjusting to external circumstances; apathy encompasses loss of interest and drive; inadequate reactions in social settings can indicate disinhibition. Behavioral impairment may be accompanied by loss of insight into disease‐related impairment in up to 25% of ALS‐FTD patients (Raaphorst et al., 2012). A consensus on what constitutes insight or loss thereof is lacking (Evers et al., 2007). Our study focused on loss of insight into disease‐related behavioral subclinical changes and clinical impairment, specifically when a patient views their own behavior differently from their primary caregiver (Woolley et al., 2010).

Such loss of impairment insight has been documented in bvFTD and ALS‐FTD (Beeldman et al., 2018; Griffin et al., 2016; Ichikawa et al., 2008; Saxon et al., 2017; Woolley et al., 2010). Woolley et al. (2010) reported that while ALS‐FTD patients viewed themselves as behaviorally impaired, they still estimated the degree of their impairment as less severe than their caregivers. ALS patients without FTD experienced mild behavioral abnormalities but retained insight (Terada et al., 2011; Woolley et al., 2010). Insight in ALS is a crucial research area, Woolley et al. (2010)’s findings have influenced the revised Strong criteria (Strong et al., 2017), the creation of the dimension apathy scale (Radakovic et al., 2016) and the ALS‐cognitive behavioural screen (Woolley et al., 2010). Current evidence only points to differences in insight between ALS‐FTD and ALS without FTD. The most recent Strong criteria profile ALS‐FTSD patients into the following subgroups: ALS without cognitive impairment (ALSni), ALS with cognitive impairment (ALSci), ALS with behavioral impairment (ALSbi), ALS with cognitive and behavioral impairment (ALScbi), and ALS‐FTD (Strong et al., 2017). Differences in insight between these profiles have not yet been investigated. A further gap in insight research in ALS is the absence of studies investigating the effect of premorbid intelligence. Retained insight with higher levels of verbal intelligence has been documented in other neurodegenerative disorders (Spitznagel & Tremont, 2005).

Our study aimed to address these gaps by profiling patients based on the Strong criteria (Strong et al., 2017), and exploring the effect of intelligence on insight into behavioral impairment. We hypothesized that ALS‐FTD and behaviorally impaired patients would self‐rate as behaviorally impaired while losing insight into their impairments’ magnitude compared with their caregivers. We also expected these patient groups to exhibit greater loss of insight into their behavioral decline than ALS patients without behavioral impairments.

## 2 METHODOLOGY

### 2.1 Participants

Eighty‐three ALS patients and their family members were recruited prospectively at outpatient clinics in Rostock and Magdeburg, Germany (for previous publications from this study, see Kasper et al., 2015; Kasper et al., 2016; Machts et al., 2014). Patients were diagnosed using the revised El Escorial criteria (Brooks et al., 2000). Twenty‐nine percent had possible ALS (*n* = 24), 28% had probable ALS (*n* = 23), 17% had definite ALS (*n *= 14), and 26% were not classifiable by the El Escorial criteria because they had a pure upper or lower motor neuron syndrome (*n* = 22). Phenotypes included classic ALS (72%, *n* = 59); predominantly upper motor neuron syndrome (4%, *n* = 3), progressive muscular atrophy (15%, *n* = 12), flail arm (2%, *n* = 2), flail leg (6%, *n* = 5), and two unknown phenotypes (2%, *n* = 2). Disease onset was spinal in 42% of cases (*n* = 35), and bulbar in 30% (*n* = 25). For 28%, discerning an onset type at time of diagnosis was not possible. All patients but one consented to genetic testing: 90% of cases were sporadic, 5% had the *SOD1* mutation (*n *= 4), 2% had the *C9orf72* mutation (*n *= 2), and one person had the *VAPB* mutation. Similarly, low frequencies of *SOD1* and *C9orf72* mutations have been documented in another Northern German sample (Krüger et al., 2016). Participants were profiled according to the Strong and Rascovsky criteria (Rascovsky et al., 2011; Strong et al., 2017) (see Table 1). There were two demographic differences (Table 1): ALSci patients had a lower verbal‐crystallized premorbid intelligence than ALSni patients (prior odds = 0.32, posterior odds = 1177.15, BF_10 _= 3684.25, error% = 9.980e‐9) and ALSbi patients (prior odds = 0.32, posterior odds = 12.02, BF_10 _= 37.61, error% = 1.765e‐5). The family informants consisted of spouses (*n* = 65, 78%), children (*n* = 9, 11%), and other relatives (*n* = 9, 11%).

**TABLE 1:** Demographic background of the sample

Measure	ALSni	ALSci	ALSbi	ALScbi	ALS‐FTD
*N* (%)	33 (40%)	12 (14%)	26 (31%)	8 (10%)	4 (5%)
Sex (f/m)	11/22***	3/9***	8/18***	2/6***	1/3***
Age	59.27 (13.77)	60.25 (11.74)*	62.00 (10.87)	61.63 (12.18)*	67.25 (7.63)
Education (Years)	12.88 (2.25)	12.25 (1.66)	13.12 (2.29)*	12.25 (2.19)	12.50 (2.65)
Premorbid IQ	101.77 (8.71)	91.20 (7.10)†	100.19 (10.42)*	97.83 (9.68)	97.67 (21.22)
ALSFRS‐R	37.66 (6.95)	37.50 (4.80)*	35.96 (8.44)	36.38 (8.44)	37.25 (10.47)
Disease duration (Months)	24.33 (19.49)	20.92 (14.93)*	27.08 (25.69)*	31.38 (23.22)	20.00 (11.40)
ALSFRS‐R δ	0.64 (0.47)	0.86 (0.86)	0.89 (1.10)	1.03 (1.18)	0.49 (0.24)

* BF_01 _> 3, moderate evidence of no differences to ALSni;

** BF_01 _> 30, very strong evidence of no differences to ALSni;

*** BF_01 _> 100, extremely strong evidence of no between‐group differences in sex distribution.

† BF^10 ^> 3, moderate evidence of a difference to ALSni.

This study was conducted in accordance with the Declaration of Helsinki and approved by each site's local ethics committee (reference numbers A2010‐32 and A2011‐56). Participants gave written, informed consent.

### 2.2 Measurements

#### 2.2.1 Frontal systems behavior scale (FrSBe)

This questionnaire features 46 items for patients and informants to rate the frequency of premorbid behaviors retrospectively, and current behaviors contemporaneously (scaled from 1 [“almost never”] to 5 [“almost always”]). Participants were instructed to consider the time before the onset of motor symptoms for the premorbid ratings and the time of testing for the current ratings. The FrSBe includes the domains of apathy, disinhibition and executive dysfunction, and a total score. Raw scores are transformed into age‐, sex‐, and education‐adjusted *T* scores for clinical interpretation; *T* ≥ 65 indicates behavioral impairment (Grace & Malloy, 2001). Based on these, we calculated insight as follows. First, we subtracted premorbid scores from current scores (“change ratings”, following Woolley, Moore et al., 2010). Then, we subtracted the informants’ change ratings from the patients’ change self‐ratings, so that a lower numerical value would indicate greater loss of insight into their behavioral decline (threshold *T*_Insight_ ≥ −20, Woolley et al. (2010)). Simultaneously, a *T*_Insight_ ≤ 20 would signify a loss of insight by which the patients over‐report, that is, exaggerate their behavioral abnormalities in comparison to their informants. Therefore, the *T*_Insight_ captures loss of insight into present clinical and subclinical changes, in addition to absent changes. The FrSBe was combined with an informant interview to distinguish between behavioral changes and behavioral impairments. If patients met the Strong criteria according to the informants’ FrSBe or the informants reported that the behavioral changes impaired patients’ ability to conduct their everyday lives, patients were considered behaviorally impaired. Our data provided evidence that there was no effect of familial relation between informant and patient on change ratings for apathy (BF_01 _= 6.20), disinhibition (BF_01 _= 5.87), executive dysfunction (BF_01 _= 5.59), and overall change (BF_01 _= 6.20).

#### 2.2.2 Premorbid IQ

Participants underwent thorough neuropsychological examination, including an estimate of verbal‐crystallized intelligence (Kasper et al., 2015; Kasper et al., 2016; Machts et al., 2014). Estimates of premorbid verbal IQ were obtained after disease onset by asking the participants to identify correct target words among distractor pseudo‐words. The number of correctly identified target words was converted to an IQ estimate based on published norms (Schmidt & Metzler, 1992). ALS patients’ performance on this specific test has been shown to remain intact (Lange et al., 2016; Neudert et al., 2001; Temp et al., 2021), and independent from physical disability (Osmanovic et al., 2020; Temp et al., 2021).

### 2.3 Statistical analysis

Prior to our primary analyses, we confirmed that behavioral decline was present using paired samples t‐tests (see Figure 2) (following Witgert et al. (2010); Woolley, Moore et al. (2010)). We further investigated whether premorbid IQ—which differed between groups—influenced insight using Kendall's tau (τ) correlation coefficient (Field et al., 2012).

Our primary analyses were analyses of covariance with cognitive‐behavioral profile according to the Strong criteria as the between‐subjects independent variable, IQ as the covariate, and *T*_Insight_ in apathy, disinhibition, executive dysfunction, and overall behavioral problems as outcomes. As we had hypothesized the cognitive subgroups of ALS to differ in insight, we aimed to support the most likely alternative hypothesis instead of rejecting the null hypothesis. To this end, we employed Bayes factor hypothesis testing which permitted us to quantify support in favor of our hypothesis by comparing it to the null model (Wagenmakers, 2007; Wagenmakers et al., 2018; Wagenmakers et al., 2018). The data were analyzed in *Jeffreys’ Amazing Statistics Program* (JASP, The JASP Team, 2019), with JASP set to report the best model atop the results table, and BF_01_ favoring this best model. Markov chain Monte Carlo sampling for numerical accuracy occurred 10,000 times; seeds were determined by icosahedron, with seeds set to 59163 (apathy), 163613 (disinhibition), 514417 (executive dysfunction), and 15112 (total). One‐tailed Mann–Whitney U tests served as post hoc tests between the behaviorally impaired and behaviorally unimpaired groups; 1000 bootstraps were used (seed = 201,416).

We applied the following evidence categories (Wagenmakers et al., 2018): a BF above 3 provides “moderate evidence”, a BF above 10 provides “strong evidence”, a BF above 30 provides “very strong evidence”, and a BF above 100 provides “extreme evidence” in favor of the best model.

## 3 RESULTS

First, we ascertained the presence of subclinical behavioral changes or clinically relevant behavioral impairment across patient groups before exploring the influence of IQ, and the differences in insight between patient groups.

### 3.1 Behavioral impairment: Premorbid versus current

**FIGURE 1:**
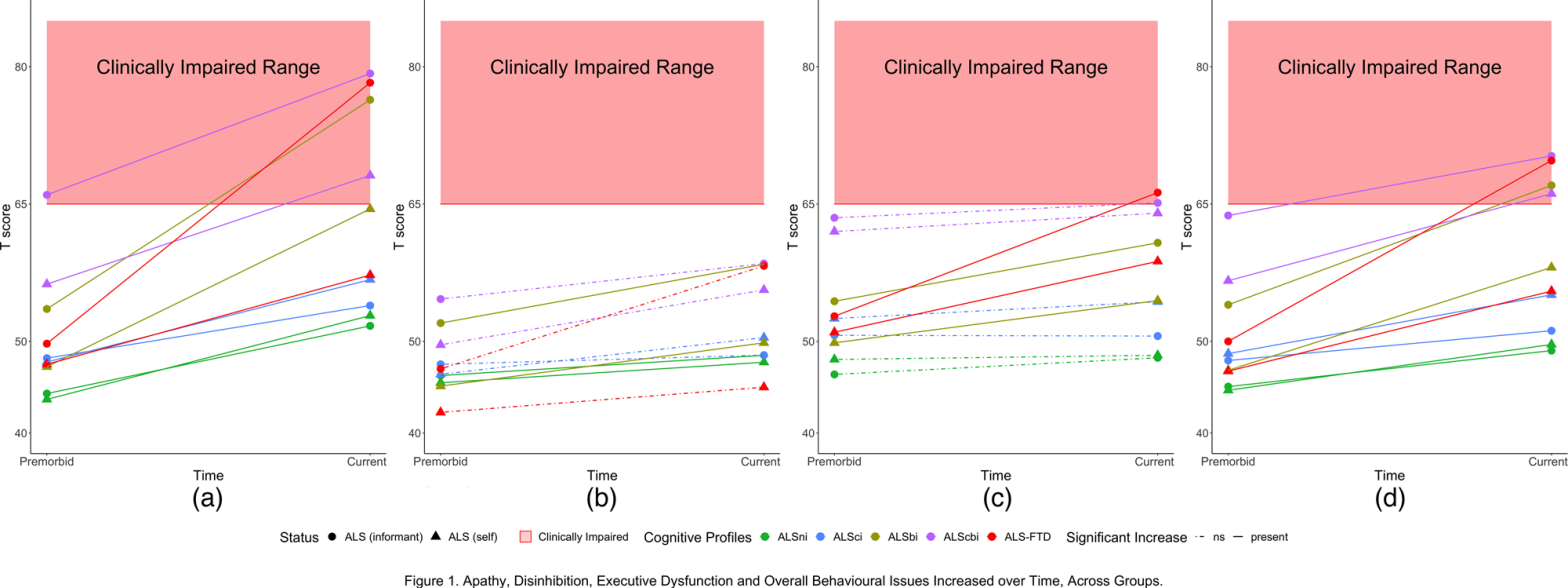
Apathy, disinhibition, executive dysfunction and overall behavioural issues increased over time, across cognitive profiles. (a) Increases in apathy ratings. (b) Increases in disinhibition ratings. (c) Increases in executive dysfunction ratings. (d) Increases in overall behavioural dysfunction ratings

In Figure 1, clinically relevant impairments (*T* ≥ 65) are depicted within the red bands and statistically significant increases in behavioral abnormalities are signified by solid lines.

### 3.2 Current self‐ratings

Clinically relevant impairments were self‐reported by the ALScbi group across the apathy and total behavioral change domains (Figure 1a,d); the ALSni, ALSci, ALSbi, and ALS‐FTD groups did not self‐report any impairments.

Statistically relevant subclinical increases in behavioral abnormality were self‐reported by all patient groups across the apathy and total behavioral change domains (Figure 1a,d). The ALSni patients further self‐reported increased disinhibition (Figure 1b), while the ALScbi patients self‐reported increased disinhibition as well as executive dysfunctioning (Figure 1b,c) and the ALS‐FTD patients self‐reported increased executive dysfunction (Figure 1c).

### 3.3 Current informant ratings

Clinically relevant impairments were reported by the informants of the ALSbi, ALScbi, and ALS‐FTD patients across the apathy and total behavioral changes domains (Figure 1a,d)—as necessitated by the Strong criteria. Additionally, the ALS‐FTD and ALScbi informants reported impairments in the executive domain (Figure 1c). Only the ALScbi patients were rated to exhibit behavioral impairment prior to motor symptom onset by their informants (Figure 1a,e).

Statistically significant increases in behavioral abnormalities were present in all subgroups. ALSni patients’ informants also reported statistically significant, subclinical increases in apathy, disinhibition, and the total behavioral change domain (Figure 1a,b,d). Similarly, ALSci patients were reported to exhibit increased behavioral abnormalities across these domains. Given the statistical evidence of increasingly abnormal behavior, we investigated insight across all profiles, including the ones where behavioral change did not correspond to clinically relevant (*T* < 65) behavioral impairment.

### 3.4 Insight

**FIGURE 2:**
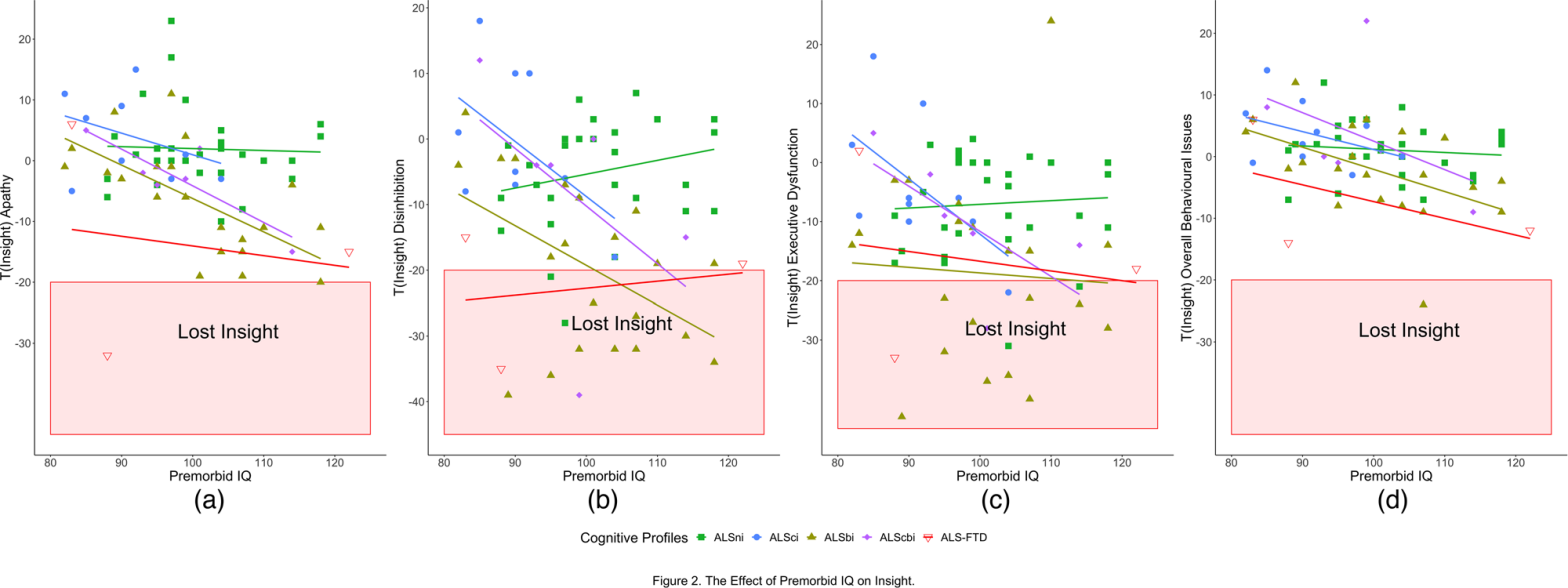
The effect of premorbid IQ on insight. (a) Higher IQ was associated with worse insight into apathy. (b) Higher IQ was associated with worse insight into disinhibition for ALSci, ALSbi and ALScbi patients, but with better insight for ALSni and ALS‐FTD patients. (c) Higher IQ was associated with worse insight into executive dysfunction in impaired patient groups, but with better insight in ALSni patients. (d) Higher IQ was associated with worse insight into overall behavioural dysfunction in all patient groups

Next, we explored whether the effect of premorbid IQ on insight differed between Strong profiles (Figure 2). Higher premorbid IQ correlated with worse insight into apathy (τ = −0.24, BF = 9.72) and overall behavioural decline (τ = −0.27, BF = 36.13), but there were no correlations with executive dysfunction (τ = −0.13, BF = 0.57) or disinhibition (τ = −0.14, BF = 0.65). For apathy, executive dysfunction and overall impairment, the effect of IQ was homogenous between Strong profiles (Figure 2a,c,d). Its effect on disinhibition, however, was heterogeneous (P(M) = 0.20, P(M|data) = 1.537e‐4, BF_M _= 6.292e‐4, BF_01 _= 3978.21, error% = 0.83; compared to the null model). ALSni and ALS‐FTD patients with a higher IQ retained better insight into disinhibition, while ALSci, ‐bi, and ‐cbi patients with a higher IQ demonstrated worse insight into disinhibition (Figure 2b). To ascertain that the variance in the ALS‐FTD was not the driving force behind these effects, the IQ‐related results were repeated and replicated without the ALS‐FTD group. Consequently, we corrected our primary analyses for premorbid IQ by including it into the null model.

For our primary analyses, we reported evidence that was at least moderately in favor, or moderately against our hypothesis; inconclusive results can be found in our online supplement. These ANCOVA test the hypothesis that patients of different Strong profiles exhibit different levels of insight into each behavioral domain by comparing the effect of Strong profile to the corrected null model, representing the effect that changes in insight are solely driven by the covariate IQ.

#### 3.4.1 Apathy

**FIGURE 3:**
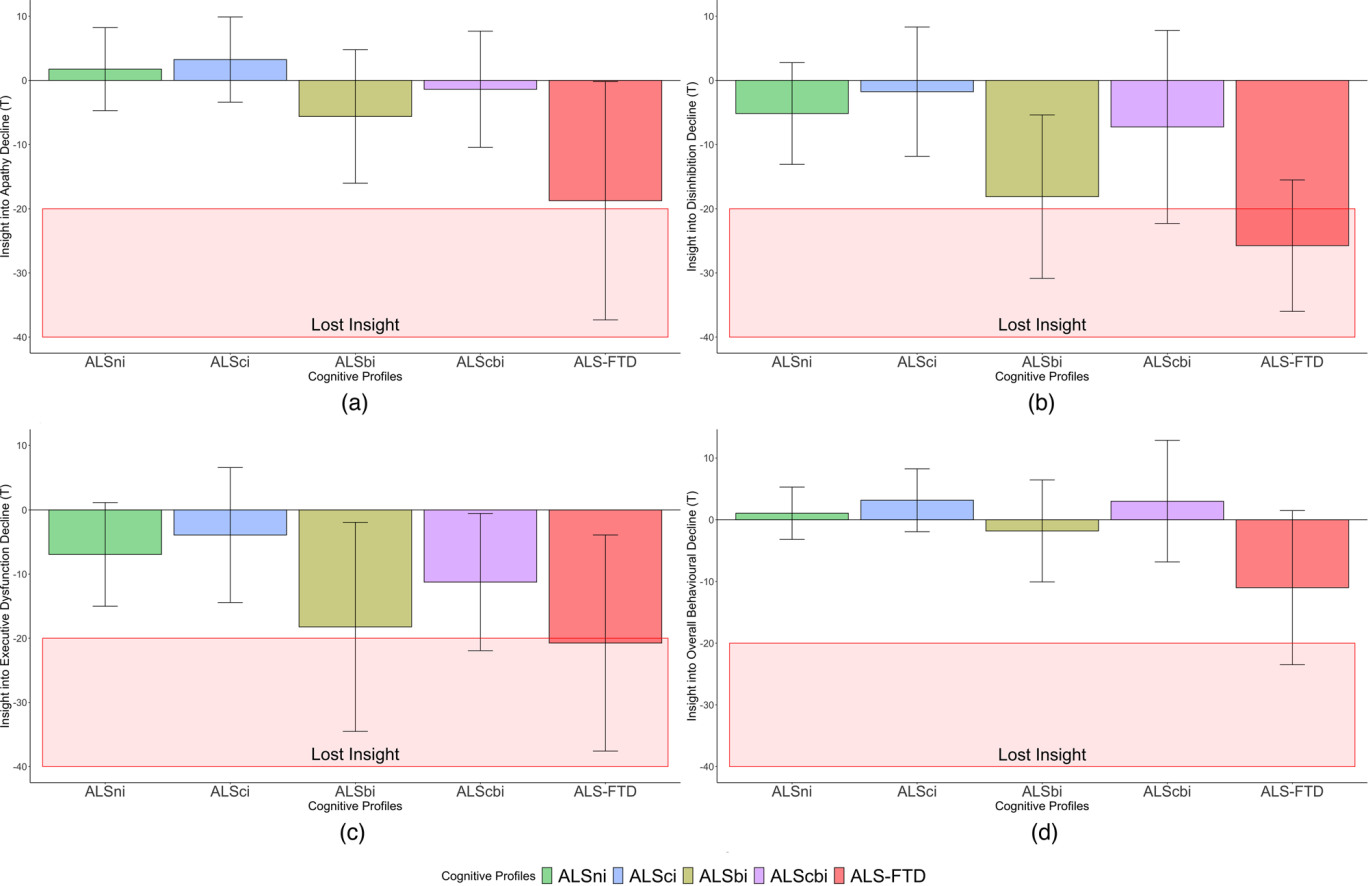
Insight into changes in apathy, disinhibition, executive dysfunction and overall behavioural decline across groups. (a) ALSbi patients retained less insight into changes in apathy than ALSni and ALSci patients. (b) ALS‐FTD patients retained worse insight into changes in disinhibition than ALSci patients, and ALSbi patients retained worse insight than ALSni patients. (c) ALSbi patients retained worse insight into changes in executive dysfunction changes than ALSni and ALSci patients. (d) There were no statistically meaningful differences in insight into overall behavioural issues

There was extremely strong evidence favoring between‐group differences in insight into apathy (P(M) = 0.50, P(M|data) = 0.01, BF_M _= 0.01, BF_01 _= 196.94, error% = 0.88). This indicates that while the null hypothesis and alternative hypothesis were considered equally probable (50%, P(M) = 0.50) prior to our data analysis, the null hypothesis was reduced to 1% and the alternative increased to 99% plausibility. There was strong evidence that ALSbi patients retained less insight into apathy than ALSni patients (BF = _−0 _= 21.78, W = 219.50, R̂ = 1.00), and moderate evidence compared to ALSci patients (BF_‐0 _= 4.31, W = 74.00, R̂ = 1.01). Evidence regarding the expected group differences between the ALS‐FTD/‐cbi patients and ALSni/‐ci patients was inconclusive; note the former's’ large standard deviations in Figure 3a. Clinically, all patient subgroups retained insight (all *T*_Insight _> −20, Figure 3a).

#### 3.4.2 Disinhibition

Evidence favoring group differences in insight into disinhibition was extremely strong, compared to the null model (P(M) = 0.50, P(M|data) = 8.106e‐4, BF_M _= 8.113e‐4, BF_01 _= 1232.58, error% = 1.01). This shows that the null hypothesis decreased in plausibility to below 0.1%, the group differences increased in plausibility to 99%.

Post hoc tests provided moderate evidence that ALS‐FTD patients retained worse insight into disinhibition than ALSci patients (BF_‐0 _= 4.32, *W* = 1.00, R̂ = 1.01). There was very strong evidence that ALSbi patients retained worse insight into disinhibition than ALSni patients (BF_‐0 _= 94.46, W = 165.50, R̂ = 1.01), and strong evidence compared to ALSci patients (BF_‐0 _= 16.74, W = 50.00, R̂ = 1.02). Evidence regarding the expected group differences between the ALScbi patients and ALSni/‐ci patients was inconclusive. Clinically, only ALS‐FTD patients lost insight into their increasing disinhibition (Figure 3b).

#### 3.4.3 Executive dysfunction

There was strong evidence favoring a between‐group difference in insight into executive dysfunction (P(M) = 0.50, P(M|data) = 0.06, BF_M _= 0.06, BF_01 _= 16.65, error% = 1.08). The null hypothesis was reduced in plausibility from 50% to 6%, and the group differences hypothesis increased in plausibility to 94%. Post hoc, there was strong evidence that ALSbi patients retained worse insight into their executive dysfunction than ALSni patients (BF‐0 = 30.67, W = 195.00, R̂ = 1.01), and moderate evidence compared to ALSci patients (BF_‐0 _= 7.27, W = 55.50, R̂ = 1.01). Evidence regarding the expected group differences between the ALS‐FTD/‐cbi patients and ALSni/‐ci patients was inconclusive. Clinically, only the ALS‐FTD patients lost insight into their declining executive functions (Figure 3c).

#### 3.4.4 Total behavioral issues

Statistically, the evidence regarding the main effect was inconclusive (P(M) = 0.50, P(M|data) = 0.55, BF_M_ = 1.20). The null hypothesis increased in plausibility from 50% to 55% while the alternative was reduced to 45%, so no clear preference between them can be established. Clinically (*T* > −20), none of the Strong profile groups lost insight into their overall behavioral decline (Figure 3d).

Our data—including the ALS‐FTD patients—provided sufficient evidence that bulbar and spinal onset patients exhibited the same levels of insight into apathy (BF = 9), executive dysfunction (BF = 5), disinhibition (BF = 8), and overall behavioral issues (BF = 4.6).

## 4 DISCUSSION

We investigated the research questions of behavioral impairment, loss of “insight” and the influence of verbal intelligence in ALS‐FTSD by conceptually replicating previous methodologies (Grossman et al., 2007; Spitznagel & Tremont, 2005; Spitznagel et al., 2006; Woolley et al., 2010). We measured behavior with instruments identical to those of Grossman et al. (2007) and Woolley, Moore et al. (2010), and we estimated verbal intelligence similar to Spitznagel and Tremont (2005) and Spitznagel et al. (2006). The original methodological contributions of this study lie in its strict profiling according to the most recent Strong criteria (Strong et al., 2017), in addition to its exploration of associations between intelligence and “insight” in ALS. Furthermore, our conceptualization of “insight” as failure to recognize subclinical behavioral changes or clinically relevant behavioral impairments which are present or absent, reduced the risk of biasing our analyses towards the behaviorally impaired patient groups.

### 4.1 Behavioral impairment

All Strong profile subgroups experienced statistically significant increases in behavioral abnormalities (Figure 1) in accordance with the literature (Raaphorst et al., 2012). In contrast to the results of Woolley et al. (2010), the ALS‐FTD patients in our study did not self‐report any clinically relevant impairments. Whereas the ALSbi patients only self‐reported a clinical impairment in the apathy domain, the ALScbi patients self‐reported clinical impairments in apathy, executive dysfunctioning, and overall behavior. According to their informants, the ALScbi patients only exhibited apathy impairments prior to motor symptom onset. This is concordant with previous findings (Grossman et al., 2007; Mioshi et al., 2014) without differentiating between Strong profiles of ALS. No patient group exhibited disinhibition (Figure 1b). Clinically relevant levels of disinhibition in ALS without FTD are subject to debate in the literature: Mioshi et al. (2014) report that 75% of ALS patients experience no or only mild symptoms of disinhibition, while Grossman et al. (2007) report that 29% exhibit impairment in this domain. In general, our results suggest that noticeable increases in behavioral abnormalities occur even in behaviorally unimpaired ALS patient groups. The impact of these changes on the patients and their families’ lives remains to be studied.

### 4.2 Insight between cognitive‐behavioral strong profiles

Our ALSni and ALSci groups retained insight into their mild behavioral abnormalities, as expected, based on previous findings (Terada et al., 2011; Woolley et al., 2010). Detailed distinctions between ALSbi and ALScbi patients, however, had been lacking. Our findings suggest that both groups retain insight clinically. Unexpectedly, our ALS‐FTD patients retained insight into their apathy impairment, meeting the clinical criterion for loss of insight (*T*_Iinsight_ ≤ −20) only in the disinhibition and executive domains, where they did not consider themselves impaired (Figures 1 and 3). This clinically relevant loss of insight occurred only in our ALS‐FTD group, congruent with previous literature (Beeldman et al., 2018; Raaphorst et al., 2012; Saxon et al., 2017; Woolley et al., 2010).

Regardless of clinically significant impairment in insight, we had expected the ALS‐FTD ALScbi and ALSbi patients to experience stronger loss of insight than the ALSci and ALSni patients. There was strong evidence of those between‐group differences in our data regarding all individual domains but not the total score. An increased loss of insight emerged primarily in the ALSbi and ALS‐FTD patients. However, evidence regarding the ALS‐FTD patients was inconclusive in the domains of apathy and executive dysfunction. There was also insufficient evidence to facilitate conclusions regarding the insight of ALScbi patients. Our results thus establish an absence of evidence, though not of effects. This absence may be explained by our small ALScbi and ‐FTD groups (*n* = 8, and *n* = 4, respectively); a larger sample would be beneficial to provide more conclusive evidence.

We replicated the loss of insight documented by Woolley et al. (2010) in ALS‐FTD but not all the differences from ALSni patients. Conceptual replications such as ours yield mixed results in approximately 10% of attempts, while 4% fail and 86% succeed (Makel et al., 2012). In addition to the small sample sizes, several methodological aspects may explain our mixed replication results. The internal coherence of our Bayesian modeling techniques ensures that there is no sample size below which our inferences could be viewed as untrustworthy (Wagenmakers et al., 2018), and our ALS‐FTD sample size (*n* = 4) was identical to that of Woolley et al. (2010). Nevertheless, the instances of inconclusive evidence in our data may be due to our sample size, and/or the heterogeneity of FTD. Rates of loss of insight in ALS‐FTD patients have been contrastive between a larger sample size taken from a meta‐analysis by Raaphorst et al.(2012) and the smaller sample sizes of both our study and that of Saxon et al. (2017): the former group observed loss of insight in only 25% of 170 ALS‐FTD patients whereas 75% was detected out of four ALS‐FTD patients in our study and 88% was described in 56 ALS‐FTD patients by Saxon et al. (2017), again suggesting that studies investigating loss of insight with larger sample sizes would be of value to reduce the risk of bias. This heterogeneity across studies suggests that the exclusion of loss of insight from the Rascovsky criteria for bvFTD (Rascovsky et al., 2011) was an improvement in sensitivity over the Neary criteria, which had included loss of insight (Neary et al., 1998).

### 4.3 Insight and premorbid verbal intelligence

Paradoxically, higher verbal intelligence was associated with worse insight into apathy and overall behavioral issues (Figure 2a,d). Group differences in intelligence affected insight into disinhibition heterogeneously (Figure 2b), with highly intelligent ALS‐FTD and ALSni patients retaining higher insight, and highly intelligent ALSci, ALSbi, and ALScbi patients exhibiting decreased insight. In all other domains, patient groups experienced a greater loss of insight with higher verbal intelligence homogeneously (Figure 2a,c,d). The result that all patients with higher intelligence experienced a lesser loss of insight has previously been described in other neurodegenerative diseases (Spitznagel & Tremont, 2005; Spitznagel et al., 2006). Three possible mechanisms may underlie our paradoxical findings. The first mechanism concerns the patients themselves: individual bvFTD patients may experience a loss of insight to different degrees in that they may perceive their disease‐related decline as a threat to themselves and their lives (Griffin et al., 2016). This perceived threat may be more pronounced in those with a higher premorbid ability, resulting in a more vicarious denial of behavioral abnormalities and thus reduced insight. The second mechanism concerns the patient–carer relationship. Given that married couples are often of similar cognitive and intelligence levels (Caillot‐Ranjeva et al., 2021; Mascie‐Taylor, 1989; Watson et al., 2004), our more intelligent participants would have similarly intelligent spouses who, in turn, may potentially have rated them more harshly than the spouses of less intelligent patients owing to their related critical thinking abilities. Third, our results may indicate either that the patients or their family members were unable to express degrees of behavioral abnormality adequately or that we, at present, lack the sufficient measurement tools. These three mechanisms are not mutually exclusive.

One limitation of this work was the very small number of ALS‐FTD patients who were still capable of self‐rating; it is conceivable that a loss of insight would have been more apparent in those who could no longer self‐rate. The study design of comparing self‐ratings to informant ratings by default introduces bias into our sample as patients who were cognitively unable to comprehend and complete the FrSBe were excluded from the analysis. Conclusive evidence was only provided in the subgroups with *n* ≥ 12. Possibly, the low sample size is at the root of the absent evidence in the smaller subgroups. However, where effects are small or heterogeneous, even large samples may produce absence of evidence.

Though the FrSBe is considered the gold standard for measuring behavioral impairments, it may overestimate them in ALS as some behavioral symptoms may indeed be related to motor impairment(s) (Pinto‐Grau et al., 2017). German translations of alternative, ALS‐specific behavioral instruments would certainly be desirable (Elamin et al., 2017; Mioshi et al., 2014; Raaphorst et al., 2012). These results would benefit from future research replicating our findings in larger cohorts, especially in ALS‐FTD and ALScbi patients. Recent efforts to establish a cognitive reserve in ALS have been successful (Canosa et al., 2020; Consonni et al., 2020; Costello et al., 2021; Temp et al., 2021), and future efforts could investigate that concept more specifically in relation to behavior and insight. For the sake of clarification, the paradox of lower levels of insight at higher levels of intelligence deserves future investigation; perhaps a more holistic approach to intelligence including the family raters would produce different results. Furthermore, the relationship between behavioral changes and caregiver burden and its implications for “insight” requires future investigation.

In conclusion, behavioral decline occurred in ALS patients regardless of their cognitive‐behavioral profiles. Only the ALScbi patients experienced behavioral impairment prior to motor onset. Insight into behavioral decline was present in all domains for all non‐demented patient groups but absent in disinhibition and executive dysfunctioning in the ALS‐FTD group. Our study presents the paradox that higher premorbid verbal intelligence was associated with poorer insight, a finding worthy of further investigation.

## CONFLICT OF INTEREST

S.J.T. declares the following (all in Germany): MSD Sharp & Dohme GmbH, Lindenplatz 1, 85549 Haar; 11/09/2018: talk “Dementia and Diabetes–current report” at the quality circle for physicians in Kühlungsborn; 14/11/2018: MSD expert forum NAB Alzheimer in Munich, participant as consultant; 13/08/2019: talk “Dementia and Diabetes–current report” at the event “Dementia and Diabetes” in Rostock; ROCHE Pharma AG, Emil‐Barell‐Str. 1, 79639 Grenzach‐Wyhlen; 12/09/2019: 3^rd^ National Advisory Board for ROCHE Pharma AG, Frankfurt (Main), participant as consultant; 27/09/2019: talk “Amyloid as target for diagnosis and treatment in Alzheimer's disease” at the ROCHE Symposium at the DGN Congress in Stuttgart; 09/2020: Advisory board Biogen, Biogen GmbH, Riedenburger Straße 7, 81677 Munich; 01/22/2021: online expert discussion “Mild Cognitive Impairment”, talk “MCI Epidemiology”, Dr. Willmar Schwabe GmbH & Co. KG, Willmar‐Schwabe‐Str. 4, 76227 Karlsruhe; 25/03/2021: virtual advisory board “National Alzheimer Advisory Board”, consultant, Roche Pharma AG, Emil‐Barell‐Str. 1, 79639 Grenzach‐Wyhlen. A.H. received royalties from BIOGEN and DESITIN for advisory board meetings.

## AUTHOR CONTRIBUTIONS

Anna G. M. Temp: Research question, data analysis design, data analysis, interpretation of results, preparation of manuscript. Elisabeth Kasper: Study design, neuropsychological data collection, interpretation of results, and review of manuscript. Stefan Vielhaber: Study design, neurological data collection, and review of manuscript. Judith Machts: Study design, neuropsychological data collection, and review of manuscript. Andreas Hermann: Study design, data analysis design, and review of manuscript. Stefan Teipel: Study design, data analysis design, and review of manuscript. Johannes Prudlo: Study design, neurological data collection, data analysis design, review of manuscript, funding acquisition, and principal investigator.

### PEER REVIEW

The peer review history for this article is available at https://publons.com/publon/10.1002/brb3.2439.

## Data Availability

A CSV file and the HTML results transcript from JASP are available on the Open Science Framework: https://osf.io/r7jz2/.
